# Distinct Tumor Microenvironments Are a Defining Feature of Strain-Specific CRISPR/Cas9-Induced MPNSTs

**DOI:** 10.3390/genes11050583

**Published:** 2020-05-23

**Authors:** Amanda Scherer, Victoria R. Stephens, Gavin R. McGivney, Wade R. Gutierrez, Emily A. Laverty, Vickie Knepper-Adrian, Rebecca D. Dodd

**Affiliations:** 1 Department of Internal Medicine, University of Iowa, Iowa City, IA 52242, USA; amanda-scherer@uiowa.edu (A.S.); victoria.r.stephens@vanderbilt.edu (V.R.S.); emily-laverty@uiowa.edu (E.A.L.); vickie-knepper@uiowa.edu (V.K.-A.); 2 Holden Comprehensive Cancer Center, University of Iowa, Iowa City, IA 52242, USA; gavin-mcgivney@uiowa.edu (G.R.M.); wade-gutierrez@uiowa.edu (W.R.G.); 3 PREP program, University of Iowa, Iowa City, IA 52242, USA; 4 Cancer Biology Graduate Program, University of Iowa, Iowa City, IA 52242, USA; 5 Medical Scientist Training Program, University of Iowa, Iowa City, IA 52242, USA

**Keywords:** CRISPR/Cas9, MPNST, mouse models, sarcoma, tumor microenvironment

## Abstract

The tumor microenvironment plays important roles in cancer biology, but genetic backgrounds of mouse models can complicate interpretation of tumor phenotypes. A deeper understanding of strain-dependent influences on the tumor microenvironment of genetically-identical tumors is critical to exploring genotype–phenotype relationships, but these interactions can be difficult to identify using traditional Cre/loxP approaches. Here, we use somatic CRISPR/Cas9 tumorigenesis approaches to determine the impact of mouse background on the biology of genetically-identical malignant peripheral nerve sheath tumors (MPNSTs) in four commonly-used inbred strains. To our knowledge, this is the first study to systematically evaluate the impact of host strain on CRISPR/Cas9-generated mouse models. Our data identify multiple strain-dependent phenotypes, including changes in tumor onset and the immune microenvironment. While BALB/c mice develop MPNSTs earlier than other strains, similar tumor onset is observed in C57BL/6, 129X1 and 129/SvJae mice. Indel pattern analysis demonstrates that indel frequency, type and size are similar across all genetic backgrounds. Gene expression and IHC analysis identify multiple strain-dependent differences in CD4+ T cell infiltration and myeloid cell populations, including M2 macrophages and mast cells. These data highlight important strain-specific phenotypes of genomically-matched MPNSTs that have implications for the design of future studies using similar *in vivo* gene editing approaches.

## 1. Introduction

Mouse models are a cornerstone of cancer research and have produced a wealth of mechanistic insights into tumor biology. While mice from a wide variety of genetic backgrounds are used for *in vivo* cancer modeling, there is strong evidence that strain-dependent phenotypes can complicate interpretation of results. Within similar genetic contexts, mouse strain can impact tumor susceptibility, disease onset, metastatic potential, and the spectrum of cancer development [1,2,3,4,5]. Multiple strain-dependent cancer phenotypes can be attributed to background-specific modifying loci [6,7]. Classic examples include tumor development in *Nf1*^+/-^; *p53*^+/-^ mice (*NPcis*), which have high incidences of astrocytomas and malignant peripheral nerve sheath tumors (MPNST) on a C57BL/6 background but are less tumor prone on other genetic backgrounds. Extensive genetic mapping experiments determined that astrocytoma susceptibility is linked to an imprinted locus on chromosome 11, while MPNST formation is associated with polymorphisms in the nerve sheath tumor resistance (*Nstr*) genes [8,9,10]. The development of neurofibromas, benign nerve sheath tumors that are precursor lesions to MPSNTs, is also strain dependent. Schwann cell-specific overexpression of neuregulin in *p53*^+/−^ mice (P_0_-GGFβ3; p53^+/−^) drives neurofibroma formation on a mixed background, but mice fail to develop tumors after backcrossing onto an inbred C57BL/6J background [11]. In addition to tumorigenesis events, metastatic phenotypes can also be dramatically influenced by genetic background, as observed in *Pten*-driven prostate cancer models [12,13] and MMTV-PyMT-driven mammary tumors [14].

Strain-dependent variations in the tumor microenvironment (TME) can also profoundly impact cancer phenotypes. The TME is comprised of a diverse array of extracellular matrix and stromal cells including cancer-associated fibroblasts, endothelial cells, and immune infiltrates. Variations in the immune systems of common inbred strains are well documented [15]. For example, C57BL/6 mice have elevated neutrophils and splenic macrophages, but decreased B cell and CD4+ T cell populations compared to BALB/c and 129/SvHsd mice [16,17]. Polarization of macrophage function is strain dependent, with enrichment of classically-activated, pro-inflammatory M1 macrophages in Th1-oriented mouse strains such as C57BL/6, while immunosuppressive M2 macrophages are predominant in Th2-oriented mouse strains such as BALB/c [18]. Functional activity of immune cells is also heavily influenced by mouse background, including the cytotoxic capacity of NK cells [19] and macrophage recruitment [20].

Multiple tumor phenotypes can be attributed to differences in host immune function, including metastatic potential and therapeutic response. Depletion of myeloid cell-derived MMP9 in MMTV-PyVT models slows metastatic progression in C57BL/6 mice, but had no impact on pulmonary metastases in an FVB/N background [21]. In syngeneic transplant models, antibody blocking experiments demonstrate that melanoma metastasis is dependent on strain-specific NK cell activity [19]. These differences in the strain-dependent immune landscape have implications for immunotherapy response in preclinical models [22,23,24,25,26]. Multiple groups have reported that while immunosuppressive cells predominate in poorly-responsive models, cytotoxic effector cells are prevalent in tumors of responsive models.

A deeper understanding of the impact of host strain background on the TME of genetically-identical tumors is necessary to help guide future experimental design and interpretation of preclinical cancer studies. The nature of genetically-engineered mouse models (GEMMs) and syngeneic cell transplant models have necessitated that data are obtained from tumors arising in a limited number of genetic contexts and tissues. Therefore, most basic and translational studies utilize only a single inbred mouse strain, and the majority of primary model studies have been conducted predominantly in C57BL/6 and 129/S mice. However, this current paradigm of using a small number of genetic backgrounds does not address the important role of TME variation as a determinant of cancer phenotype.

The development of somatic CRISPR/Cas9 tumorigenesis approaches allows for direct comparisons of host TME in genetically-identical tumors. We have recently published a CRISPR/Cas9-induced model of soft-tissue sarcoma in wild-type mice [27]. This approach delivers an adenovirus expressing Cas9 and guide RNAs targeting *Nf1* and *p53* into the sciatic nerve of adult mice to generate high-fidelity malignant peripheral nerve sheath tumors (MPNSTs), a high-grade sarcoma of the myelinating nerve sheath. This system allows for introduction of multiple somatic mutations into adult animals surrounded by native, non-mutant stroma and an intact immune system. By introducing somatic gene alterations into adult mice without the need for lengthy and costly backcrossing, CRISPR/Cas9 approaches can assess genetic events in different murine backgrounds. Because this approach uses exogenous delivery of Cas9, it can be applied to a mouse from any strain or pre-existing genetically-engineered model. This adaptability is important to facilitate studies that rely on specific strains for experimental models, such as in the fields of metabolic disease and immunology.

To our knowledge, a systematic study examining the impact of host strain on CRISPR/Cas9-generated mouse models has not been undertaken. Here, we use CRISPR/Cas9 approaches to determine the influence of mouse background on genetically-identical MPNSTs. We report variations in tumor onset, immune landscape, and TME-associated gene expression across MPNSTs generated in four classically inbred strains. These data highlight important strain-specific phenotypes of genomically-matched MPNSTs that have implications for the future design of studies using similar in vivo gene editing approaches. Ultimately, CRISPR/Cas9 tumorigenesis approaches may provide unique opportunities to explore TME-dependent events by leveraging the diversity of stromal landscapes across tumor models from distinct genetic backgrounds.

## 2. Materials and Methods

### 2.1. Animals

All animal experiments were performed in accordance with protocols approved by the University of Iowa Institutional Animal Care and Use Committee (IACUC) and adhere to the NIH Guide for the Care and Use of Laboratory Animals. C57BL/6 (stock #556) and BALB/c mice (stock #555) were purchased from Charles River Laboratories. 129X1 mice (stock #000691) were purchased from Jackson Laboratories. Wild-type 129Sv/Jae mice were bred and maintained at the University of Iowa.

### 2.2. CRISPR/Cas9 Generated MPNSTs and Growth Analysis

Adenovirus containing Cas9 and sgRNAs targeting *Nf1* and *p53* was purchased from ViraQuest (North Liberty, Iowa) [27]. Prior to injection, virus was mixed with DMEM and calcium phosphate as previously described [28,29,30]. Tumors were generated by injection of 25 uL of prepared virus into the left sciatic nerve of mice. When tumors reached a volume of 150 mm^3^ (Day 1), they were measured by calipers 3 times weekly. Tumor volumes were calculated using the formula *V* = (*π* × *L* × *W* × *H*)/6, with *L, W*, and *H* representing the length, width, and height of the tumor in mm, respectively. Tumors were harvested when they reached a volume of 1500 mm^3^ or earlier if animals showed signs of distress, in accordance with IACUC guidelines at the University of Iowa. Tissue was collected for histology, RNA, and generation of cell lines. 

### 2.3. Generation of Cell Lines from MPNSTs 

Cell lines were derived from terminally-harvested MPNSTs. Tumors were finely minced and digested in dissociation buffer Collagenase Type IV (700 units/mL, Thermo, 17104-019, Thermo Fisher Scientific, Waltham, MA, USA) and dispase (2.4 units/mL, Thermo, 17105-041, Thermo Fisher Scientific, Waltham, MA, USA) in PBS for 1–1.5 h at 37 °C on an orbital shaker. Dissociated tissue was passed through a sterile 70 µM cell strainer (Fisherbrand, 22363548, Thermo Fisher Scientific, Waltham, MA, USA), washed once with PBS, and resuspended in DMEM (Gibco, 11965-092, Thermo Fisher Scientific, Waltham, MA, USA). Cells were cultured in DMEM containing 10% FBS, 1% penicillin-streptomycin (Gibco, 15140-122, Thermo Fisher Scientific, Waltham, MA, USA) and 1% sodium pyruvate (Gibco, 11360-070, Thermo Fisher Scientific, Waltham, MA, USA). After 10 passages, cells were used for indel analysis and subsequent studies.

### 2.4. Indel Analysis

Indel pattern analysis was previously described [31]. Genomic regions of *Nf1* and *p53* that spanned the gRNA target sites were amplified by PCR using Phusion high-fidelity DNA polymerase (NEB, M0530L). PCR primers for *Nf1* indels generate a 569 bp fragment in wild-type cells while those used to amplify *p53* indels result in a 520 bp fragment in wild-type cells. Primer sequences are listed in Supplementary Table S1. PCR amplicons were purified with the Monarch PCR and DNA Cleanup Kit (NEB T1030S). Sanger sequencing was performed by the Genomics Division of the Iowa Institute of Human Genetics at the University of Iowa. Indel frequencies were quantified from the chromatograms by sequence trace analysis using Synthego ICE [32]. Indels > 50 bp were determined by band size on a 2% agarose gel.

### 2.5. Histology and Immunohistochemistry

Upon harvest, a portion of tumor tissue was stored in 10% neutral buffered formalin for fixation and subsequent paraffin embedment. Formalin-fixed paraffin embedded tumors were sectioned and stained with hematoxylin (Vector Laboratories, H-3401, Burlingame, CA, USA) and eosin (Sigma-Aldrich, 586-X, St. Louis, MO, USA) to evaluate tissue morphology. All immunostaining was conducted with citrate-based antigen retrieval (Vector Laboratories, H-3300, Burlingame, CA, USA). The following antibodies were used: S100 (Abcam, ab4066, Cambridge, United Kingdom), Ki67 (BD Biosciences, 556003), CD4 (Abcam, ab183685), CD8a (Thermo Fischer Scientific, 14-0808-82, Waltham, MA, USA), Foxp3 (Thermo Fisher Scientific, 14-4777-82, Waltham, MA, USA), and F4/80 (Thermo Fisher Scientific 14-4801-82, Waltham, MA, USA). To visualize mast cells, slides were stained with toluidine blue solution (0.02% toluidine blue in 1% NaCl, pH 2.2) for 2 min, followed by two washes in distilled water and three washes in 100% ethanol. At least five tumors per group were analyzed, and quantification of cells staining positive was performed on 6 independent fields. The 20× fields were used for all analyses except for Ki67, which used 40× fields. Imaging was performed using an EVOS XL Core Imaging System (Thermo Fisher Scientific, AMEX1000, Waltham, MA, USA).

### 2.6. Quantitative RT-PCR

Upon harvest, tumor tissue was stored in RNA Later (AM7020, Thermo Fisher Scientific) at −20 °C. Tumors (*n* = 5 per strain) were homogenized in liquid nitrogen and resuspended in Trizol (15596018, Thermo Fisher Scientific, Waltham, MA, USA). cDNA was synthesized from 1 ug of RNA using iScript (1708891, Bio-Rad). RT-qPCR was performed with Power-up Sybr Green 2x Master Mix (A25778, Thermo Fisher Scientific, Waltham, MA, USA) per the manufacturer’s instructions on an Applied Biosystems 7900HT instrument using the ΔΔC_t_ relative to B2M expression (Genomics Division of the Iowa Institute of Human Genetics, University of Iowa). Primer sequences are listed in Supplementary Table S1 [24].

### 2.7. Statistical Analysis

Statistical analysis was performed using GraphPad Prism 8. Tumor growth kinetics, IHC quantification, and gene expression were analyzed using a one-way ANOVA with Tukey’s multiple comparison test. Sample sizes for IHC and qRT-PCR analysis were 5 per group. Comparison of survival curves was performed using the log-rank (Mantel–Cox) test. For all studies, a *p* value of less than 0.05 was considered statistically significant.

## 3. Results

### 3.1. Host Strain Determines Tumor Onset for Genetically-Identical MPNSTs

**Figure 1:**
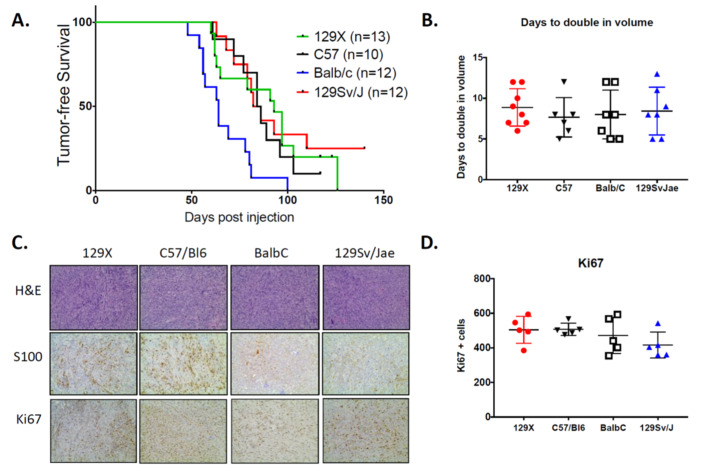
Host strain determines tumor onset but does not alter tumor growth kinetics. (**A**) Kaplan–Meyer curve of tumor-free survival. Formation of *Nf1/p53*-deleted malignant peripheral nerve sheath tumors (MPNSTs) is accelerated in BALB/c mice. Tumor initiation occurs within a similar timeframe in mice from 129/SvJae, C57BL/6, and 129X1 backgrounds. (**B**) Growth kinetics are similar across all background strains for genetically-identical MPNSTs (*n*= 6–8 tumors per strain). Growth rates are calculated as the number of days required for tumors to double from an initial volume of 150 mm^3^. 129X1 (red circles), C57BL/6 (black triangles), BABL/c (white squares), and 129/SvJae (blue triangles). (**C**) Representative images of MPNSTs from different host strains stained for H&E (20×), S100 (20×), and Ki67 (20×). (**D**) Quantification of Ki67 confirms that background strain does not alter the rate of tumor proliferation (*n*= 5 tumors per strain). (**B**,**D**) analyzed by one-way ANOVA with Tukey’s multiple comparison test.

To determine the impact of murine background strain on MPNST development, we generated somatic CRISPR/Cas9-induced tumors in four commonly-used laboratory strains: 129/SvJae, C57BL/6, 129X1, and BALB/c. Importantly, the 129/SvJae mice serve as reference controls, as this strain was used in our prior study [27]. We injected the sciatic nerve of 10–13 mice per background with adenovirus containing Cas9 and guide RNAs for *Nf1* and *p53* (Ad-Cas9 + gNF1 + gp53). This approach was previously shown to generate high-fidelity, *Nf1*/*p53*-null MPNSTs at the site of injection within 3–4 months. Similar to our prior data, 129/SvJae mice in the current study develop tumors at ~80 days post-injection (Figure 1A). Tumor onset is similar in C57BL/6 and 129X1 mice, arising at an average of 82 and 93 days, respectively. In contrast, BALB/c mice develop MPNSTs earlier than other strains, with tumors developing with an average onset of 61 days. After tumor detection, MPNSTs were measured 3x/weekly to obtain proliferative rates, which are calculated from a uniform initiating size of 150 mm^3^. The average time for tumors to double in volume is 7–8 days, which is similar across all backgrounds (Figure 1B). Tumor proliferation was also examined by immunohistochemistry for Ki67 in terminally-harvested MPNSTs. Ki67 indices are similar in tumors from all strains, supporting the observation that host strain does not influence MPNST proliferation (Figure 1C). Histological analysis confirms MPNST morphology in all tumors, with S100 positivity noted in tumors from each background (Figure 1D). Taken together, these data show that somatic CRISPR/Cas9 tumorigenesis approaches can generate MPNSTs in a broad spectrum of wild-type mice, and that background strain can influence tumor initiation in genetically-matched tumors.

### 3.2. Indel Analysis Reveals Unique Patterns of Gene Disruption

**Figure 2:**
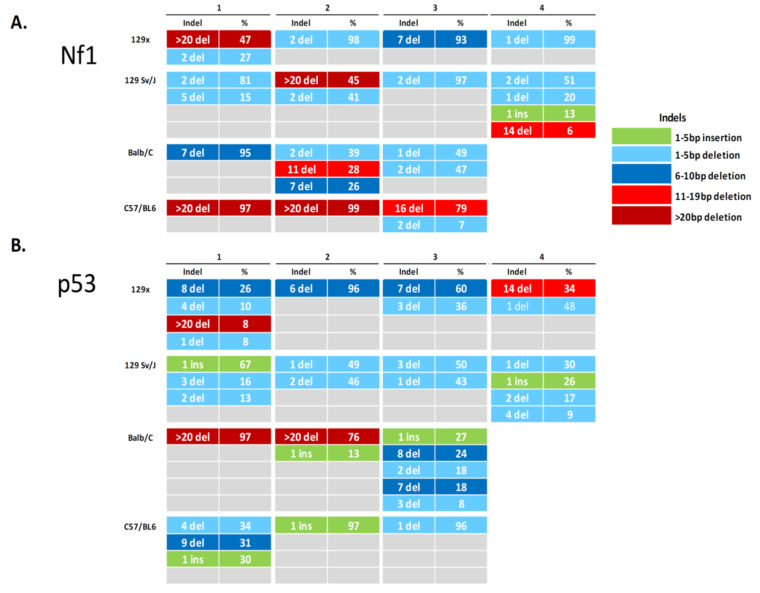
CRISPR/Cas9-induced insertions and deletions detected in *Nf1* and *p53* in MPNST-derived cell lines from different genetic backgrounds. Indel pattern analysis of the sgRNA-targeted regions of *Nf1* (**A**) and *p53* (**B**) demonstrates disruption of genomic targets in all tumors. The majority of indels detected in both *Nf1* and *p53* are frameshift mutations that result in inactivation of targeted proteins.

Indel signatures can determine the spectrum and frequency of CRISPR/Cas9-induced events in individual tumors. We generated tumor-derived cell lines to evaluate the unique indel patterns within each MPNST (Figure 2). Our analysis confirms the presence of *Nf1* and *p53* indels in all tumors. Additionally, no wild-type sequence is detectable in any cell line, suggesting complete disruption of the targeted regions. As CRISPR/Cas9 generates indels by random reassembly of DNA, we investigated the types of indels generated with each guide RNA. To focus this analysis, we evaluated indels that occur at > 5% frequency. Across 14 tumor-derived cell lines, we observe 24 indels in *Nf1* and 33 indels in *p53*. Several cell lines have a simple signature, containing predominantly one indel, while others have complex signatures comprised of up to five distinct variants per gene. The majority of cell lines contain multiple *p53* indels, as a single dominant indel of *p53* is detected in only 4/14 (29%) of cells. Single indels in *Nf1* are more frequent, with 7/14 (50%) of cell lines containing a solitary *Nf1* indel event. Insertions are less common than deletions, with only 1/14 (7%) of cell lines harboring *Nf1* insertions and 6/14 (43%) of cell lines harboring *p53* insertions. Indeed, only one cell line does not have a deletion event in *p53*, with a single predominant insertion being the only indel event detected within the sample. In our analysis, CRISPR-generated insertions are genetically small (1–2 bp), while deletions occur within a larger range (1 bp to > 20 bp). In *p53* indels, we observe a trend towards smaller deletions (<10 bp), which occur in 23/27 (85%) of deletion events. All of the indels detected in *Nf1* were either frameshift (FS) mutations (20/24) or indels ≥ 20 bp (4/24) that are the most likely to disrupt protein function by shifting the reading frame and inducing premature termination, nonsense mediated decay (NMD), or alterations in protein structure [33,34]. For indels detected in *p53*, 24/33 were FS mutations and 3/33 were deletions ≥ 20 bp. We did not identify any strain-specific trends in indel type, size, or frequency in this analysis, suggesting that in vivo CRISPR/Cas9 genomic editing occurs similarly across different murine backgrounds.

### 3.3. Immunological Diversity of MPNSTs Is a Hallmark of Genetic Background 

Data from genetically-engineered mouse models strongly support a role for host strain in distinct patterns of immune cell activation [18,24]. Therefore, we hypothesized that there are strain-dependent differences in the composition of the immune landscape in our CRISPR/Cas9 generated MPNSTs. To examine the tumor microenvironment in genetically-identical tumors from different mouse strains, we performed histological analysis for populations of innate and adaptive immune cells that play key roles in MPNST biology, including CD4+ T cells, CD8+ T cells, regulatory T lymphocytes (Tregs), macrophages and mast cells in five tumors per genetic background (Supplementary Figure S1). 

**Figure 3:**
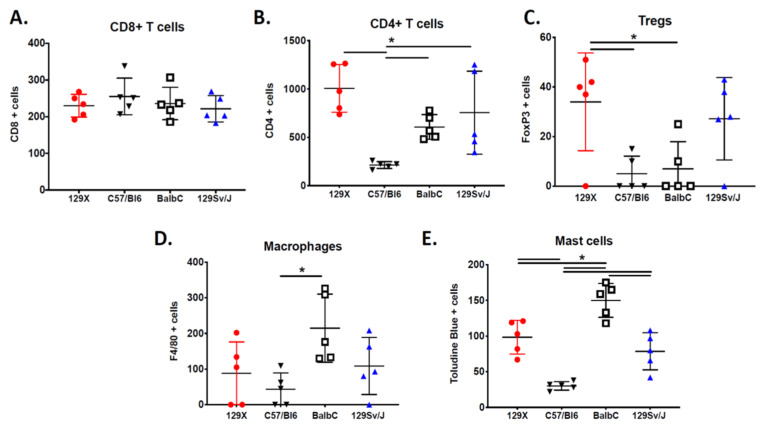
The MPNST immune landscape is determined by genetic background. (**A**) Levels of CD8+ T cells in terminally-harvested MPNSTs are similar across all host strains. (**B**) Infiltration of CD4+ T cells are significantly lower in tumors from C57BL/6 mice compared to MPNSTs in mice from 129X1, BALB/c, and 129/SvJae backgrounds. (**C**) Foxp3+ Tregs are detected at higher levels in tumors from 129X1 mice compared to C57BL/6 and BALB/c mice. (**D**) MPNSTs from BALB/c mice have significantly higher levels of infiltrating F4/80+ macrophages compared to C57BL/6 mice. (**E**) Mast cell infiltration is higher in tumors from BALB/c mice compared to 129/SvJae, C57BL/6, and 129X1 mice. Mast cell levels are lowest in MPNSTs from C57BL/6 mice. 129X1 (red circles), C57BL/6 (black triangles), BABL/c (white squares), and 129/SvJae (blue triangles). Analyzed by one-way ANOVA with Tukey’s multiple comparison test. A *p*-value of less than 0.05 is considered statistically significant and is denoted by “*” (*n* = 5 tumors per strain).

Levels of tumor-infiltrating cytotoxic CD8+ T lymphocytes are similar across all host strains (Figure 3A). In contrast, amounts of CD4+ T lymphocytes are highly dependent on background strain, with MPNSTs from C57BL/6 mice having lower CD4+ T infiltration than tumors on 129Sv/Jae, BALB/c, and 129X1 backgrounds (Figure 3B). MPNSTs from 129Sv/Jae mice display a heterogenous distribution of CD4+ T lymphocytes, with a wide variability of cell number across individual tumors. Regulatory T cells levels are highly variable across individual tumors, most likely due to the rare nature of these cells. In several tumors, we were unable to detect a single Treg in the sample. Analysis of multiple tumors determined that MPNSTs from 129X1 mice have higher levels of Tregs than MPNSTs from C57BL/6 or BALB/c mice (Figure 3C). Analysis of macrophage levels by F4/80 staining shows increased macrophage infiltration in MPNSTs from BALB/c mice (Figure 3D). Mast cells, histamine-rich myeloid cells with a strong role in MPNST biology [30,35], are enriched in MPNSTs from BALB/c mice (Figure 3E). The lowest levels of mast cells are observed in tumors from C57BL/6 mice. Taken together, these observations demonstrate the broad diversity of immune landscapes in MPNSTs from different background strains. 

### 3.4. Gene Expression of the MPNST Microenvironment

**Figure 4:**
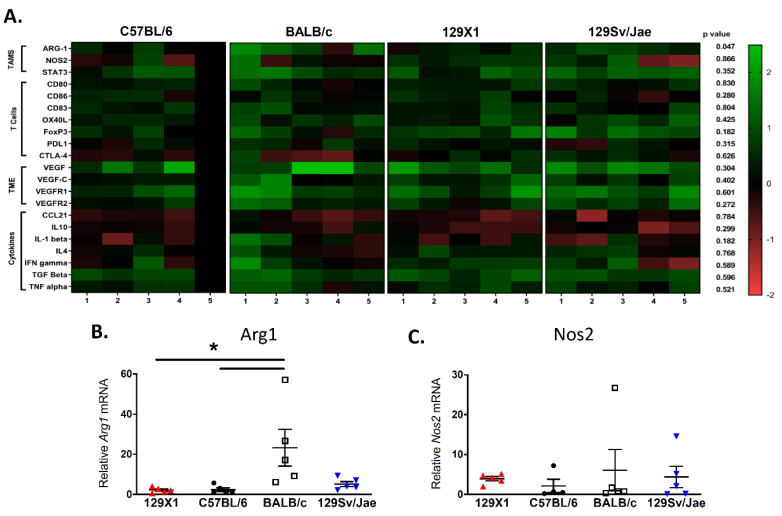
Expression of key genes in the MPNST microenvironment. (**A**) RT-qPCR analysis of markers for innate immunity, adaptive immunity, angiogenesis, and cytokine signaling in terminally-harvested tumors shows a large degree of heterogeneity between host strains and individual tumors. Samples are normalized to a single C57BL/6 tumor, shown as reference (*n* = 5 tumors per strain). (**B**) Expression analysis determines that MPNSTs from BALB/c mice express significantly higher levels of *Arg-1* mRNA, a marker of immunosuppressive M2 macrophages, when compared to tumors from 129/SvJae, C57BL/6, and 129X1 mice. (**C**) In contrast, levels of *Nos2* mRNA, a marker of M1 macrophages, is similar in tumors from all background strains. 129X1 (red circles), C57BL/6 (black triangles), BABL/c (white squares), and 129/SvJae (blue triangles). Analyzed by one-way ANOVA with Tukey’s multiple comparison test. A *p*-value of less than 0.05 is considered statistically significant and is denoted by “*”.

Given the broad variability of strain-dependent immune infiltration observed in our IHC data, we chose to perform extensive gene expression analysis of key tumor microenvironmental markers [24]. Using real-time qPCR analysis of whole tumor lysates from five tumors per background, we evaluated expression levels of pathways involved in innate immunity, adaptive immunity, angiogenesis, and cytokine signaling (Figure 4A and Supplementary Figure S2). These data provide insight into key tumor–stroma interactions and reveal extensive heterogeneity across host strains and individual tumors.

We first examined expression of tumor-associated macrophage (TAM) genes, since they are one of the most differentially-regulated immune cell populations between host strains. Expression of *Arg1* mRNA, a marker of immunosuppressive M2 macrophages, is elevated in MPNSTs from BALB/c mice (Figure 4B). Of note, *Arg1* is the only gene in our analysis that is statistically different between host backgrounds (*p* = 0.0156, one-way ANOVA). There were no differences in levels of the M1 macrophage marker *iNos1/Nos2* in tumors from different host strains (Figure 4C), suggesting that the influx of macrophages in MPNSTs from BALB/c mice consists of *Arg1*-expressing TAMs of the M2 subtype. This finding is consistent with data demonstrating that expression of the pro-immunogenic, M1 macrophage transcription factor *Stat3* is similar across backgrounds.

To further explore T lymphocyte populations, we examined genes involved in T cell activation and signaling. Expression of APC-resident co-stimulatory molecules—including *CD80*, *CD86*, *OX40L*, and *PDL1*—are similar across host strains. Similarly, expression of *CTLA-4*, an inhibitory receptor that negatively regulates T cell responses, and *CD83*, a marker of activated CD4+ T lymphocytes and dendritic cells, is not strain dependent. Levels of the regulatory T cell marker *FoxP3* are not statistically different across strains due to extensive heterogeneity between tumors, although trends are similar to IHC findings in Figure 3.

We next examined expression of angiogenesis genes, including *Vegf*, *Vegfr1*, and *Vegfr2*, in addition to the lymphangiogenic growth factor *Vegfc*. While several individual tumors display high expression of these growth factors, there are no statistically significant differences between host strains. Finally, we examined expression of key cytokines involved in immune activation, including proinflammatory molecules (*Tnfa*, *Ifng*, *IL4*, *IL1b*, and *Ccl21*) and immune-suppressive cytokines (*IL10* and *Tgfb*). Several cytokines have similar expression across all tumors, including *Tnfa*, *Ifng*, and *Ccl21*. Other cytokines (including *Tgfb*, *IL4*, *IL10*, and *IL1b*) display more variability across individual tumors, although this was not associated with specific background strains. Taken together, this gene expression analysis highlights key strain-dependent differences in the composition of the tumor microenvironment—most notably, the elevation of M2 macrophages in MPNSTs from BALB/c mice.

## 4. Discussion

The genetic background of murine cancer models can determine critical phenotypes such as disease onset, metastatic potential, immune response, and treatment outcome. To examine the impact of mouse strain on the biology of genetically-identical tumors, we used somatic CRISPR/Cas9 tumorigenesis approaches to generate MPNSTs in four commonly-used, classically inbred strains. We evaluated the influence of mouse strain on tumor growth, histology, indel pattern, immune cell infiltration, and expression of TME markers. Our data indicate that background strain impacts tumor latency, immune composition, and gene expression of genetically-identical MPNSTs. In particular, BALB/c mice exhibit multiple strain-dependent tumor phenotypes, including acceleration of tumor onset, elevated mast cell infiltration, and enrichment of M2 macrophages. In contrast, MPNSTs generated in C57BL/6 mice display decreased levels of T lymphocytes. Taken together, these data highlight the importance of considering host strain in the design and interpretation of tumor studies.

CRISPR/Cas9 approaches can facilitate the study of cancer-relevant questions that are difficult to address using conventional Cre/loxP methods. The requirement for complex backcrossing and the potential for persistent modifier loci with traditional GEMM approaches complicates data interpretation, and it has been challenging to examine the impact of background strain on the immune landscape of genetically-matched tumors. While multiple groups have reported broad immunological diversity in different syngeneic cell transplant models generated within the same background strain [22,23,24,25,26], our data identify multiple strain-specific differences in tumor infiltration by myeloid and adaptive immune cells in isogenic MPNSTs. Of note, tumors from C57BL/6 mice have the lowest levels of infiltrating CD4+ T lymphocytes. This observation is in line with published work examining the immune microenvironment in a series of cell transplant models from C57BL/6 and BALB/c mice. One study found that CD4+ T lymphocytes comprise only 1–4% of total CD45 cells in syngeneic C57BL/6 models—including MC38, LL/2, and B16F10 tumors—while populations of CD4+ T lymphocytes account for 6–10% of total immune cells in syngeneic BALB/c models such as CT26, RENCA, and 4T1 [22]. We also observed increased Tregs by IHC analysis in MPNSTs from 129X1 mice. However, it is difficult to compare our findings to the other 129-derived tumor models, as there are few published studies that include 129-based models in cross-strain analysis of immune infiltration.

Our data also found enrichment of mast cells in MPNSTs from BALB/c mice. Increased mast cell levels are associated with accelerated onset of MPNSTs in *Nf1* haploinsufficient mouse models [30]. In neurofibromas, *Nf1*^+/-^ mast cells are essential to tumor formation due to critical SCF-mediated interactions with *Nf1*^+/-^ Schwann cells [35]. Indeed, mast cells may play tumor promoting roles in multiple cancers—including colorectal and pancreatic—by supporting an immunosuppressive microenvironment or altering ECM homeostasis [36]. However, the prognostic significance of mast cells varies greatly across different cancer types. While a mechanistic role for mast cells in MPNST development has not been shown, a study in a small number of patient samples (*n* = 34) found that mast cell density did not correlate with patient survival [37]. Mast cell function is strain dependent, with bone marrow-derived mast cells (BMMCs) from BALB/c mice displaying more robust responses than BMMCs from other backgrounds. For example, in response to allergenic challenge, BMMCs from BALB/c mice degranulate more efficiently [38], produce higher amounts of newly-synthesized mediators [39], and infiltrate more rapidly into bronchial tissue than BMMCs from C57BL/6 mice [40]. This increased activity of mast cells in BALB/c mice, combined with elevated mast cell infiltration in BALB/c-derived MPNSTs, could partially explain the accelerated tumor onset phenotype in this strain.

One of the strongest strain-dependent immune phenotypes we observed was enrichment of macrophages in MPNSTs from BALB/c mice. In syngeneic tumor models, macrophage infiltration is highly variable and is more dependent upon cancer type than host strain [22,23]. For example, macrophages account for ~18% of total CD45+ immune cells in both RENCA (BALB/c hosts) and Lewis Lung carcinomas (C57BL/6 hosts), while macrophages make up only ~5% of immune cells in CT26 (BALB/c hosts) and B16 melanoma (C57BL/6 hosts) models [22]. Our data also identify a strong M2 polarization in TAMs from BALB/c-derived tumors by upregulation of *Arg1* expression. This strain-specific enrichment of M1/M2 macrophages is a well-documented phenotype. As M2 macrophages predominantly promote wound healing and tissue homoeostasis, the M1/M2 polarization can have important phenotypic consequences. For example, in response to challenge with *Leishmania*, C57BL/6 mice can eliminate infection by activation of an M1/Th1 response, but BALB/c mice succumb to infection due to the inability of their M2 macrophages to mount an effective response [18].

It is important to note that the M1/M2 definition of macrophages represents a phenotypic spectrum, rather than a binary characterization. The strict definition of M1 vs. M2 has recently been broadened with the discoveries of in vivo populations that exist along a mixed M1/M2/monocyte spectrum that support plasticity among myeloid populations [41]. Indeed, macrophage diversity is widespread among mouse models, as demonstrated with data from the hybrid mouse diversity panel (HMDP) that was developed to examine immunological variation across different host backgrounds. By using a panel of 83 inbred mouse strains, this resource can perform gene association studies to better understand and map complex traits [42]. A genome-wide study of peritoneal macrophage transcriptomes from the HMDP identified a natural spectrum of macrophage activation phenotypes and confirmed that the M1/M2 axis is a major macrophage polarization phenotype in vivo [43]. Of particular importance to cancer biology, the M1 and M2 paradigm of macrophage polarization does not clearly apply to TAMs, which are strongly influenced by tumor location and external cues from the surrounding microenvironment [44]. TAM subsets can express both M1 and M2 markers simultaneously, suggesting that they display a more complex activation scenario than the simple M1/M2 activation status [41,44,45]. Nonetheless, an appreciation of strain-dependent macrophage polarity is important for interpretation and design of in vivo tumor models examining macrophage tumor biology.

One interesting observation from our study is the acceleration of tumor initiation in BALB/c mice. Several groups have reported accelerated tumor formation in *p53*^+/-^ BALB/c mice in comparison to C57BL/6 mice [2,3,4]. However, these studies did not induce spatially-restricted tumors in adult mice. One possible explanation for earlier tumor onset of *Nf1/p53*-driven MPNSTs in BALB/c mice is their strain-specific mutation in *Ink4a* (also known as *p16*). *Ink4a* is a member of the *Cdkn2a* locus that is fundamental to cell cycle entry and progression [46]. The *Cdkn2a* (*Ink4a/Arf*) allele is a well-documented example of a strain-dependent genetic variant that can impact cancer progression [47,48]. Indeed, the increased susceptibility of BALB/c mice for various cancer types has been linked to the presence of a hypomorphic *Ink4a* allele caused by mutations in the promoter region [48]. Since disruptions in *Cdkn2a* are commonly observed in clinical MPNST samples, we postulate that acceleration of tumor onset in BALB/c mice may be partially due to disruption of this locus.

These studies underscore the need to use a diverse toolkit of mouse backgrounds in cancer biology, as the reliance on single strain studies can be a barrier to a robust understanding of cancer progression [49]. We believe there is immense strength in applying a broad diversity of in vivo models to better account for the large interindividual variation of immune systems across human populations [50,51]. Additionally, these data suggest that caution must be taken in interpretation of preclinical studies, with respect to potential influences of complex, strain-specific interactions between the TME and tumor cells. Further studies are necessary to determine whether strain-specific immune landscapes would alter therapeutic outcomes in preclinical MPNST models. It is plausible that enrichment of either T lymphocytes or macrophages could alternatively impact immunotherapy response. However, chemotherapy outcomes may be less dependent upon immune composition, as we reported that murine MPNSTs with distinct myeloid cell compositions respond similarly to doxorubicin/ifosfamide-containing regimens [30]. Taken together, our findings highlight how CRISPR/Cas9 tumorigenesis approaches can provide new experimental opportunities to leverage the immunological diversity of inbred mouse strains to reveal new features of the tumor microenvironment that drive MPNST progression.

## Supplementary Materials

The following are available online at https://www.mdpi.com/2073-4425/11/5/583/s1, Table S1: Primer and guide RNA Sequences, Figure S1: IHC of innate and adaptive immune cells in CRISPR/Cas9-generated MPNSTs. Macrophages (F4/80 staining; 40×) and mast cells (toludine blue staining; 20×) are enriched in MPNSTs from BABL/c mice. Cytotoxic T cells (CD8 staining; 20×) are similar across all strains. Helper T cells (CD4 staining; 20×) are enriched in 129X1 and 129Sv/Jae tumors. Regulatory T cells (FoxP3 staining, 40×) are enriched in 129X1 tumors, Figure S2: Quantitative RT-PCR data from heatmap. Expression levels of genes in the MPNST microenvironment examining macrophages (A), adaptive immunity (B–H), angiogenesis and lymphangiogenesis (I–L), and cytokines (M–S).

## References

[1] Reilly K.M. (2016). The Effects of Genetic Background of Mouse Models of Cancer: Friend or Foe?. Cold Spring Harb. Protoc..

[2] Kuperwasser C., Hurlbut G.D., Kittrell F.S., Dickinson E.S., Laucirica R., Medina D., Naber S.P., Jerry D.J. (2000). Development of spontaneous mammary tumors in BALB/c p53 heterozygous mice. A model for Li-Fraumeni syndrome. Am. J. Pathol..

[3] Koch J.G., Gu X., Han Y., El-Naggar A.K., Olson M.V., Medina D., Jerry D.J., Blackburn A.C., Peltz G., Amos C.I. (2007). Mammary tumor modifiers in BALB/cJ mice heterozygous for p53. Mamm. Genome Off. J. Int. Mamm. Genome Soc..

[4] Blackburn A.C., Hill L.Z., Roberts A.L., Wang J., Aud D., Jung J., Nikolcheva T., Allard J., Peltz G., Otis C.N. (2007). Genetic mapping in mice identifies DMBT1 as a candidate modifier of mammary tumors and breast cancer risk. Am. J. Pathol..

[5] Brandt L.P., Albers J., Hejhal T., Pfundstein S., Gonçalves A.F., Catalano A., Wild P.J., Frew I.J. (2018). Mouse genetic background influences whether HrasG12V expression plus Cdkn2a knockdown causes angiosarcoma or undifferentiated pleomorphic sarcoma. Oncotarget.

[6] Dragani T.A. (2003). 10 years of mouse cancer modifier loci: Human relevance. Cancer Res..

[7] Dietrich W.F., Lander E.S., Smith J.S., Moser A.R., Gould K.A., Luongo C., Borenstein N., Dove W. (1993). Genetic identification of Mom-1, a major modifier locus affecting Min-induced intestinal neoplasia in the mouse. Cell.

[8] Reilly K.M., Loisel D.A., Bronson R.T., McLaughlin M.E., Jacks T. (2000). Nf1;Trp53 mutant mice develop glioblastoma with evidence of strain-specific effects. Nat. Genet..

[9] Reilly K.M., Tuskan R.G., Christy E., Loisel D.A., Ledger J., Bronson R.T., Smith C.D., Tsang S., Munroe D.J., Jacks T. (2004). Susceptibility to astrocytoma in mice mutant for Nf1 and Trp53 is linked to chromosome 11 and subject to epigenetic effects. Proc. Natl. Acad. Sci. USA.

[10] Reilly K.M., Broman K.W., Bronson R.T., Tsang S., Loisel D.A., Christy E.S., Sun Z., Diehl J., Munroe D.J., Tuskan R.G. (2006). An imprinted locus epistatically influences Nstr1 and Nstr2 to control resistance to nerve sheath tumors in a neurofibromatosis type 1 mouse model. Cancer Res..

[11] Brosius S.N., Turk A.N., Byer S.J., Brossier N.M., Kohli L., Whitmire A., Mikhail F.M., Roth K.A., Carroll S.L. (2014). Neuregulin-1 overexpression and Trp53 haploinsufficiency cooperatively promote de novo malignant peripheral nerve sheath tumor pathogenesis. Acta Neuropathol. (Berl.).

[12] Chen M.-L., Xu P.-Z., Peng X., Chen W.S., Guzman G., Yang X., Di Cristofano A., Pandolfi P.P., Hay N. (2006). The deficiency of Akt1 is sufficient to suppress tumor development in Pten+/- mice. Genes Dev..

[13] Wang S., Gao J., Lei Q., Rozengurt N., Pritchard C., Jiao J., Thomas G.V., Li G., Roy-Burman P., Nelson P.S. (2003). Prostate-specific deletion of the murine Pten tumor suppressor gene leads to metastatic prostate cancer. Cancer Cell.

[14] Lifsted T., Le Voyer T., Williams M., Muller W., Klein-Szanto A., Buetow K.H., Hunter K.W. (1998). Identification of inbred mouse strains harboring genetic modifiers of mammary tumor age of onset and metastatic progression. Int. J. Cancer.

[15] Sellers R.S., Clifford C.B., Treuting P.M., Brayton C. (2012). Immunological variation between inbred laboratory mouse strains: Points to consider in phenotyping genetically immunomodified mice. Vet. Pathol..

[16] Hensel J.A., Khattar V., Ashton R., Ponnazhagan S. (2019). Characterization of immune cell subtypes in three commonly used mouse strains reveals gender and strain-specific variations. Lab. Investig. J. Technol. Methods Pathol..

[17] Chen J., Harrison D.E. (2002). Quantitative trait loci regulating relative lymphocyte proportions in mouse peripheral blood. Blood.

[18] Mills C.D., Kincaid K., Alt J.M., Heilman M.J., Hill A.M. (2000). M-1/M-2 macrophages and the Th1/Th2 paradigm. J. Immunol. Baltim. Md 1950.

[19] Foerster F., Boegel S., Heck R., Pickert G., Rüssel N., Rosigkeit S., Bros M., Strobl S., Kaps L., Aslam M. (2018). Enhanced protection of C57 BL/6 vs. Balb/c mice to melanoma liver metastasis is mediated by NK cells. Oncoimmunology.

[20] White P., Liebhaber S.A., Cooke N.E. (2002). 129 × 1/SvJ mouse strain has a novel defect in inflammatory cell recruitment. J. Immunol. Baltim. Md 1950.

[21] Martin M.D., Carter K.J., Jean-Philippe S.R., Chang M., Mobashery S., Thiolloy S., Lynch C.C., Matrisian L.M., Fingleton B. (2008). Effect of ablation or inhibition of stromal matrix metalloproteinase-9 on lung metastasis in a breast cancer model is dependent on genetic background. Cancer Res..

[22] Mosely S.I.S., Prime J.E., Sainson R.C.A., Koopmann J.-O., Wang D.Y.Q., Greenawalt D.M., Ahdesmaki M.J., Leyland R., Mullins S., Pacelli L. (2017). Rational Selection of Syngeneic Preclinical Tumor Models for Immunotherapeutic Drug Discovery. Cancer Immunol. Res..

[23] Yu J.W., Bhattacharya S., Yanamandra N., Kilian D., Shi H., Yadavilli S., Katlinskaya Y., Kaczynski H., Conner M., Benson W. (2018). Tumor-immune profiling of murine syngeneic tumor models as a framework to guide mechanistic studies and predict therapy response in distinct tumor microenvironments. PLoS ONE.

[24] Lechner M.G., Karimi S.S., Barry-Holson K., Angell T.E., Murphy K.A., Church C.H., Ohlfest J.R., Hu P., Epstein A.L. (2013). Immunogenicity of murine solid tumor models as a defining feature of in vivo behavior and response to immunotherapy. J. Immunother. (Hagerstown Md.: 1997).

[25] Grasselly C., Denis M., Bourguignon A., Talhi N., Mathe D., Tourette A., Serre L., Jordheim L.P., Matera E.L., Dumontet C. (2018). The Antitumor Activity of Combinations of Cytotoxic Chemotherapy and Immune Checkpoint Inhibitors Is Model-Dependent. Front. Immunol..

[26] De Luca R., Neri D. (2018). Potentiation of PD-L1 blockade with a potency-matched dual cytokine-antibody fusion protein leads to cancer eradication in BALB/c-derived tumors but not in other mouse strains. Cancer Immunol. Immunother. CII.

[27] Huang J., Chen M., Whitley M.J., Kuo H.-C., Xu E.S., Walens A., Mowery Y.M., Van Mater D., Eward W.C., Cardona D.M. (2017). Generation and comparison of CRISPR-Cas9 and Cre-mediated genetically engineered mouse models of sarcoma. Nat. Commun..

[28] Dodd R.D., Añó L., Blum J.M., Li Z., Van Mater D., Kirsch D.G. (2015). Methods to generate genetically engineered mouse models of soft tissue sarcoma. Methods Mol. Biol. Clifton NJ.

[29] Dodd R.D., Mito J.K., Eward W.C., Chitalia R., Sachdeva M., Ma Y., Barretina J., Dodd L., Kirsch D.G. (2013). NF1 deletion generates multiple subtypes of soft-tissue sarcoma that respond to MEK inhibition. Mol. Cancer Ther..

[30] Dodd R.D., Lee C.-L., Overton T., Huang W., Eward W.C., Luo L., Ma Y., Ingram D.R., Torres K.E., Cardona D.M. (2017). NF1+/- Hematopoietic Cells Accelerate Malignant Peripheral Nerve Sheath Tumor Development without Altering Chemotherapy Response. Cancer Res..

[31] Maresch R., Mueller S., Veltkamp C., Öllinger R., Friedrich M., Heid I., Steiger K., Weber J., Engleitner T., Barenboim M. (2016). Multiplexed pancreatic genome engineering and cancer induction by transfection-based CRISPR/Cas9 delivery in mice. Nat. Commun..

[32] Synthego ICE v2 CRISPR Analysis Tools. https://www.synthego.com/products/bioinformatics/crispr-analysis.

[33] Lindeboom R.G.H., Supek F., Lehner B. (2016). The rules and impact of nonsense-mediated mRNA decay in human cancers. Nat. Genet..

[34] You K.T., Li L.S., Kim N.-G., Kang H.J., Koh K.H., Chwae Y.-J., Kim K.M., Kim Y.K., Park S.M., Jang S.K. (2007). Selective Translational Repression of Truncated Proteins from Frameshift Mutation-Derived mRNAs in Tumors. PLoS Biol..

[35] Staser K., Yang F.-C., Clapp D.W. (2010). Mast cells and the neurofibroma microenvironment. Blood.

[36] Rigoni A., Colombo M.P., Pucillo C. (2015). The Role of Mast Cells in Molding the Tumor Microenvironment. Cancer Microenviron. Off. J. Int. Cancer Microenviron. Soc..

[37] de Vasconcelos R.A.T., Guimarães Coscarelli P., Vieira T.M., Noguera W.S., Rapozo D.C.M., Acioly M.A. (2019). Prognostic significance of mast cell and microvascular densities in malignant peripheral nerve sheath tumor with and without neurofibromatosis type 1. Cancer Med..

[38] Nagashima M., Koyanagi M., Arimura Y. (2019). Comparative Analysis of Bone Marrow-derived Mast Cell Differentiation in C57BL/6 and BALB/c Mice. Immunol. Investig..

[39] Noguchi J., Kuroda E., Yamashita U. (2005). Strain difference of murine bone marrow-derived mast cell functions. J. Leukoc. Biol..

[40] Pae S., Cho J.Y., Dayan S., Miller M., Pemberton A.D., Broide D.H. (2010). Chronic allergen challenge induces bronchial mast cell accumulation in BALB/c but not C57BL/6 mice and is independent of IL-9. Immunogenetics.

[41] Laviron M., Boissonnas A. (2019). Ontogeny of Tumor-Associated Macrophages. Front. Immunol..

[42] Bennett B.J., Farber C.R., Orozco L., Kang H.M., Ghazalpour A., Siemers N., Neubauer M., Neuhaus I., Yordanova R., Guan B. (2010). A high-resolution association mapping panel for the dissection of complex traits in mice. Genome Res..

[43] Buscher K., Ehinger E., Gupta P., Pramod A.B., Wolf D., Tweet G., Pan C., Mills C.D., Lusis A.J., Ley K. (2017). Natural variation of macrophage activation as disease-relevant phenotype predictive of inflammation and cancer survival. Nat. Commun..

[44] Martinez F.O., Gordon S. (2014). The M1 and M2 paradigm of macrophage activation: Time for reassessment. F1000prime Rep..

[45] Murray P.J., Allen J.E., Biswas S.K., Fisher E.A., Gilroy D.W., Goerdt S., Gordon S., Hamilton J.A., Ivashkiv L.B., Lawrence T. (2014). Macrophage activation and polarization: Nomenclature and experimental guidelines. Immunity.

[46] Sherr C.J. (2001). The INK4a/ARF network in tumour suppression. Nat. Rev. Mol. Cell Biol..

[47] Mock B.A., Krall M.M., Dosik J.K. (1993). Genetic mapping of tumor susceptibility genes involved in mouse plasmacytomagenesis. Proc. Natl. Acad. Sci. USA.

[48] Zhang S., Ramsay E.S., Mock B.A. (1998). Cdkn2a, the cyclin-dependent kinase inhibitor encoding p16INK4a and p19ARF, is a candidate for the plasmacytoma susceptibility locus, Pctr1. Proc. Natl. Acad. Sci. USA.

[49] Sittig L.J., Carbonetto P., Engel K.A., Krauss K.S., Barrios-Camacho C.M., Palmer A.A. (2016). Genetic Background Limits Generalizability of Genotype-Phenotype Relationships. Neuron.

[50] Tsang J.S., Schwartzberg P.L., Kotliarov Y., Biancotto A., Xie Z., Germain R.N., Wang E., Olnes M.J., Narayanan M., Golding H. (2014). Global analyses of human immune variation reveal baseline predictors of postvaccination responses. Cell.

[51] Casanova J.-L., Abel L. (2004). The human model: A genetic dissection of immunity to infection in natural conditions. Nat. Rev. Immunol..

