# Supplementary material for: Distinct Tumor Microenvironments Are a Defining Feature of Strain-Specific CRISPR/Cas9-Induced MPNSTs
Source: Genes (Basel). 2020 May 23;11(5):583. doi: 10.3390/genes11050583 (PMC7288323; doi:10.3390/genes11050583)
Supplement: Supplementary file 1 [file genes-11-00583-s001.pdf]

Table S1: Primer and guide RNA sequences

Primer/gRNA Name	Sequence (5' to 3')	Use
gNf1	GGACATCTCCAAGGATGTGG**TGG**	gRNA targeting Nf1
gTrp53	GATGGTAAGGATAGGTCGG**CGG**	gRNA targeting Trp53
Nf1 fw	TAGCAATTTTGGGGGAACGC	PCR for Indel Analysis
Nf1 rv	GTCAGAGCCTTTCAGTCATG	
Trp53 fw	TGCCGAACAGGTGGAATATC	PCR for Indel Analysis
Trp53 rv	CATCCTGACTGTGTGTAAACTAGGCCCC	
Arg1 fw	AGAGATTATCGGAGCGCCTT	RT-qPCR
Arg1 rv	TTTTTCCAGCAGACCAGCTT	
Nos2 fw	CACTTGGATCAGGAACCTGAAGCCC	RT-qPCR
Nos2 rv	CTTTGTGCTGGGAGTCATGGAGCCG	
Stat3 fw	CAATACCATTGACCTGCCGAT	RT-qPCR
Stat3 rv	GAGCGACTCAAACTGCCCT	
Cd80 fw	CTCTTTGTGCTGCTGATTCG	RT-qPCR
Cd80 rv	GGCAAGGCAGCAATACCTTA	
Cd86 fw	CTTACGGAAGCACCCATGAT	RT-qPCR
Cd86 rv	TCTCCACGGAAACAGCATCT	
Cd83 fw	GCTCTCCTATGCAGTGTCCTG	RT-qPCR
Cd83 rv	TCGAAGGAGCTGTTTTGCTT	
OX40L (Tnfsf4) fw	CTAAGGCTGGTGGTCTCTGG	RT-qPCR
OX40L (Tnfsf4) rv	CTTTGGATTGGAGGGTCCTT	
Foxp3 fw	TGGCAGAGAGGTATTGAGGG	RT-qPCR
Foxp3 rv	CTCGTCTGAAGGCAGAGTCA	
PD-L1 (CD274) fw	GCTCCAAAGGACTTGTACGTG	RT-qPCR
PD-L1 (CD274) rv	TGATCTGAAGGGCAGCATTTC	
Ctla-4 fw	GCCTTCTAGGACTTGGCCTT	RT-qPCR
Ctla-4 rv	CACTGAAGGTTGGGTCACCT	
Vegfa fw	GCTTCCTACAGCACAGCAGA	RT-qPCR
Vegfa rv	AATGCTTTCTCCGCTCTGAA	

VEGFR1 (Flt1) fw	CTGCTCGGGTGTCTGCTT	RT-qPCR
VEGFR1 (Flt1) rv	AAGAGAGTCTGGCCTGCTTG	
VEGFR2 (Kdr) fw	AAACCTCCTGCAAGCAAATG	RT-qPCR
VEGFR2 (Kdr) rv	TCCAGAATCCTCTTCCATGC	
Ccl21 fw	CCCTGCTTCAACCATTACATCTGC	RT-qPCR
Ccl21 rv	CCTGCTGTCTCCTTCCTCATTCC	
Il10 fw	ATCGATTTCTCCCCTGTGAA	RT-qPCR
Il10 rv	TGTCAAATTCATTCATGGCCT	
Il1b fw	GAAATGCCACCTTTTGACAGTG	RT-qPCR
Il1b rv	CTGGATGCTCTCATCAGGACA	
Il4 fw	GGATGTGCCAAACGTCCTC	RT-qPCR
Il4 rv	GAGTTCTTCTTCAAGCATGGAG	
Ifng fw	TCTGGAGGAACTGGCAAAAG	RT-qPCR
Ifng rv	TTCAAGACTTCAAAGAGTCTGAGG	
Tgfb1 fw	GGAGAGCCCTGGATACCAAC	RT-qPCR
Tgfb1 rv	CAACCCAGGTCCTTCCTAAA	
Tnfa fw	CTTCTGTCTACTGAACTTCGGG	RT-qPCR
Tnfa rv	CACTTGGTGGTTTGCTACGAC	
B2M fw	GGTCTTTCTGGTGCTTGTCTC	RT-qPCR
B2M rv	GTTCAGTATGTTCGGCTTCCC	

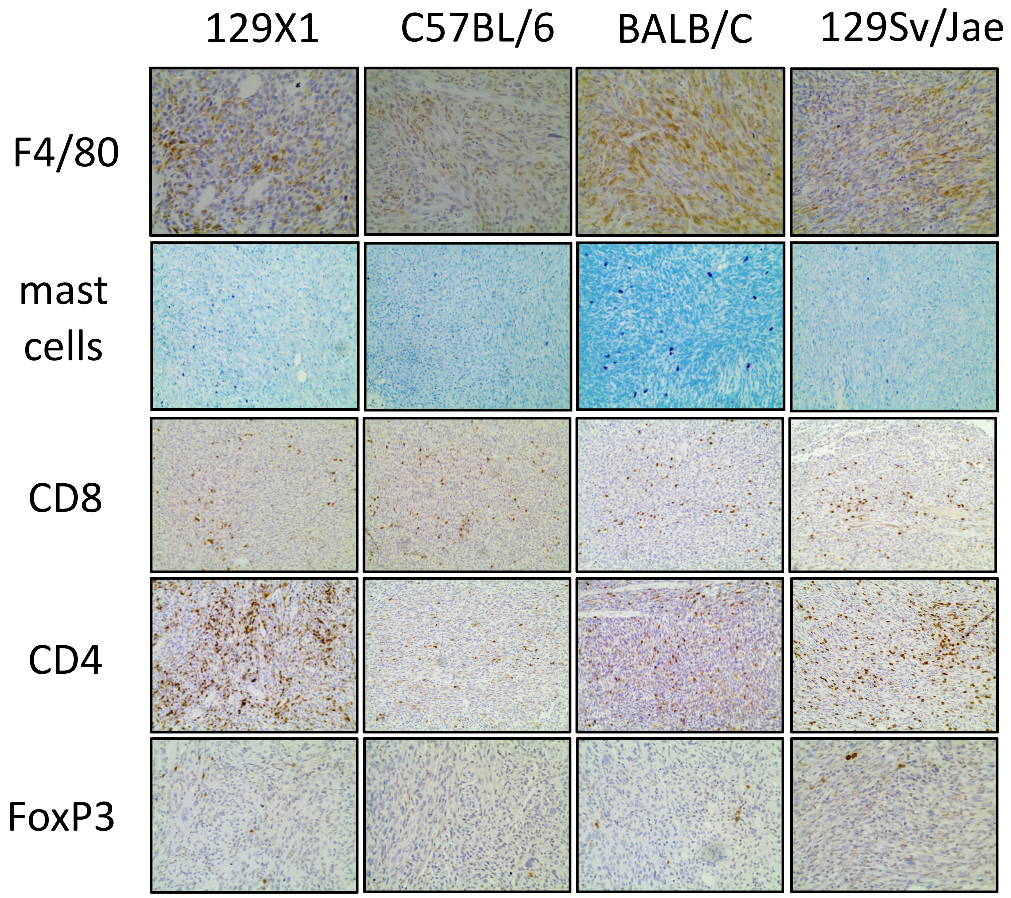

<u>Supplemental Figure 1: IHC of innate and adaptive immune cells in CRISPR/Cas9-generated MPNSTs.</u> Macrophages (F4/80 staining; 40x) and mast cells (toludine blue staining; 20x) are enriched in MPNSTs from BABL/c mice. Cytotoxic T cells (CD8 staining; 20x) are similar across all strains. Helper T cells (CD4 staining; 20x) are enriched in 129X1 and 129Sv/Jae tumors. Regulatory T cells (FoxP3 staining, 40x) are enriched in 129X1 tumors.

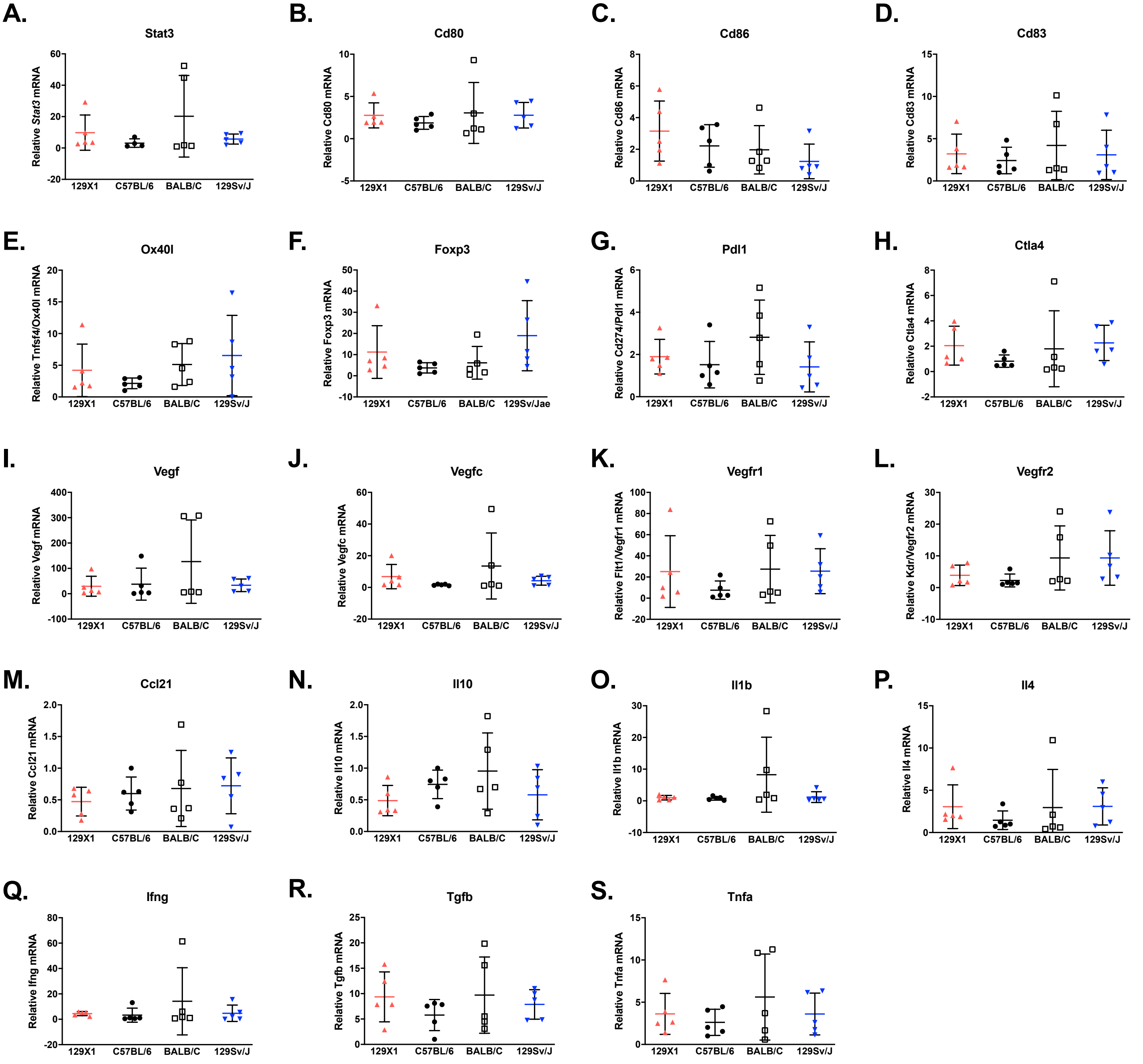

<u>Supplemental Figure 2: Quantitative RT-PCR data from heatmap.</u> Expression levels of genes in the MPNST microenvironment examining macrophages (A), adaptive immunity (B-H), angiogenesis and lymphangiogenesis (I-L), and cytokines (M-S).
